# Commentary: Intentional Observer Effects on Quantum Randomness: A Bayesian Analysis Reveals Evidence Against Micro-Psychokinesis

**DOI:** 10.3389/fpsyg.2018.01350

**Published:** 2018-08-03

**Authors:** Hartmut Grote

**Affiliations:** School of Physics and Astronomy, Cardiff University, Cardiff, United Kingdom

**Keywords:** quantum observation, micro-psychokinesis, model of pragmatic information, random number generation, RNG

In the article Maier et al. (2018), the authors report on a mind-matter experiment comprising an impressive 12,571 subjects in a Micro-PK task. The main result of that study, testing the aggregate sum of the data against its expectation value, is reported as strong evidence against Micro-Psychokinesis (PK). Despite this conclusion, the authors interpret a post-hoc observed pattern in their data as possible evidence for PK, albeit of a different kind. The authors put forward the hypothesis that a higher frequency of slow data variations can be observed in their experiment data than in a single set of control data. This commentary analyses this claim and concludes that the variation in the data motivating this hypothesis would show up just by chance with a probability of *p* = 0.328 under a null hypothesis. This author concludes that there is no evidence for the hypothesis of faster data variations, and thus for this kind of suggested Micro-Psychokinesis in the reported experimental data.

In their *post-hoc* investigation the authors of Maier et al. (2018) propose that PK-Effects show up not in the aggregate sum, but in fluctuations (i.e., the time sequence) of their data: *Interestingly, there seems to be a pattern of repeated change*. The authors connect this possible observation with theories of von Lucadou and others (e.g., von Lucadou, 2006; von Lucadou et al., 2007; von Lucadou, 2015), extended by their own thoughts about possible decline effects in Parapsychology experiments. It seems to this author that there is a confusion here about decline of the primary effect size and a decline as observed in a cumulative z-score representation of data, as used by the authors. A constant oscillation of an original effect size always leads to a decline of oscillations in a cumulative z-score plot, as more data goes into the z-score calculation. Likewise, this confusion is also evident in Figure 7 of Maier et al. (2018). The cumulative z-scores in those figures have a constant oscillation amplitude, which is only possible with an oscillating and exponentially growing effect size in the original data.

Setting this commentary aside, the authors state that: *We propose that the data presented in this study here also follow a similar systematic pattern of decline matching dampened harmonic oscillation function as suggested by Maier and Dechamps (in press)*. The authors then move to the hypothesis that their experimental data does show faster variations than expected by chance, thus supporting the existence of a particular form of PK-Effect. To support this hypothesis the authors generate one set of control data, comprising the same amount of data as the experimental data set (i.e., 12,571 “simulated” participants). They state: *Comparing Human and Simulated Data The human and the simulated data should—if the harmonic oscillation assumption is true–differ mainly in the frequency parameter* ω*. Real effects should produce more pronounced oscillations than artificial data. To explore this, we compared the 95%-confidence intervals for both frequency scores and found indeed that they did not overlap*.

This last finding, however, does not say much about the hypothesis of the authors. Deriving any significance from this single observation is incorrect. The 95%-confidence intervals of the fits of the oscillating functions have nothing to do with the question whether there is a predominant frequency (of whatever quantity) in one dataset versus another one. To illustrate this point one can imagine a single data set which is mostly composed of two different frequencies of almost equal amplitude. A fitting algorithm may find both solutions to be reasonable fits, within some error margin for each fit. However, the decision which of the two fits is actually “better” can be a marginal one. What is required in the case the authors want to assess, is not only the comparison to one control data set, but to an ensemble of *many* control data sets.

The generation of a large amount of control data sets does not necessarily have to be performed using the original apparatus, which may be a too time-consuming enterprise. Random control data can be generated with deterministic algorithms in cases the statistical parameters of the resembled experiment are sufficiently simple and well-known. Certainly this is the case for the experiment here, where only the sum of 100 binary decisions constitute one datapoint per participant. Another way to generate large amounts of control data is the use of permutations of the original experimental data or control data, obtained with the original apparatus.

For illustration, this author has generated 1,000 data sets from random permutations of the 12,571 data points of the experimental data as reported in Maier et al. (2018). Using Mathematica for the fitting of an oscillatory decaying function (the same function as noted on page 7 in Maier et al., 2018), for each data set the fit was initiated with the following start values: a = 1, *β* = 0, and p, m, and h unspecified. The start value for the frequency parameter *ω* was chosen at random for each fit on the interval 0.0005 < *ω* < 0.005, which is a range of frequencies of interest to test the hypothesis of the authors. The frequency value *ω* is the variable under test.

The fit results are shown in Figure 1 for the parameter *ω* in form of a scatter plot. To assess the quality of each fit, the variance of the fit residuals has been evaluated for each fit and is plotted as associated parameter.

**Figure 1:**
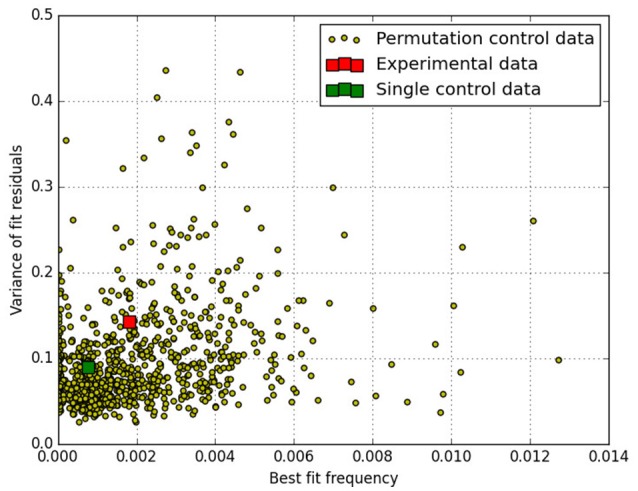
Scatter plot of 1,000 sets of permutated data that each have been fitted with the (decaying) oscillation to determine the best fit frequency (*ω*). The main experimental result as presented in Maier et al. (2018) lies at *ω* = 0.0018 with 386 results of the simulated data having larger *ω*-values than that. The majority of fits has better goodness-of-fit (smaller variance of the fit residuals) than the experimental data as reported in Maier et al. (2018). The (unpermuted) experimental data is shown as the red square and the single control data set of Maier et al. (2018) is shown as a green square.

The main experimental result as presented in Maier et al. (2018) lies at *ω* = 0.0018 (red square) with 386 results of the 1,000 simulated data sets having larger *ω*-values than that. If one ignores fits with variance larger than 0.14 (the variance of the original data), 282 out of 858 results have higher frequencies than the experimental data. In other words, the variation in the data motivating the hypothesis under test, would show up just by chance with a probability of *p* = 0.328 under a null hypothesis. This result has also been confirmed using the single control data set (green square) for the permutations, and also using a pseudo-random number generator, generating 200 complete sets of simulated data. All these simulations confirm that this result is robust with respect to the source of randomness.

Given this result, this author concludes that there is no statistical evidence for the hypothesis that the experimental data reported in Maier et al. (2018) show faster oscillations than expected by chance. Random permutations of the original data as well as new simulated data produce many data sets with higher dominant frequencies.

## Author contributions

The author confirms being the sole contributor of this work and approved it for publication.

### Conflict of interest statement

The author declares that the research was conducted in the absence of any commercial or financial relationships that could be construed as a potential conflict of interest.
